# Development and Standardization of a High-Throughput *Bordetella pertussis* Growth-Inhibition Assay

**DOI:** 10.3389/fmicb.2020.00777

**Published:** 2020-04-23

**Authors:** Anaïs Thiriard, Dominique Raze, Camille Locht

**Affiliations:** Univ. Lille, CNRS, Inserm, CHU Lille, Institut Pasteur de Lille, U1019 – UMR 9017 – CIIL – Center for Infection and Immunity of Lille, Lille, France

**Keywords:** *Bordetella*, growth-inhibition assay, antibody, complement, antibiotic

## Abstract

*Bordetella pertussis*, the main causative agent of whooping cough, is a reemerging pathogen, and recent vaccine-resistant strain outbreaks and emergence of macrolides-resistant strains in China raised new concerns for control of the disease. New vaccines and potentially new antibiotics are thus needed. *B. pertussis* is tedious to culture and requires several days of growth to count isolated colonies on agar-based media, making large-scale screening of new anti-*B. pertussis* compounds or functional evaluation of large sample sizes of immune sera difficult. Here, we developed a scalable, rapid, high-throughput luminescence-based *Bordetella* growth inhibition assay (BGIA) to quantify surviving bacteria after treatment with anti-*B. pertussis* compounds. A strong correlation between luminescence and colony-forming units (r^2^ = 0.9345, *p* < 0.0001) was found and the BGIA showed high sensitivity and reproducibility. We demonstrate here that the BGIA can be used to quantify resistance of *B. pertussis* to antibiotics, sensitivity to complement and to human serum in an easy-to-operate and fast manner. We have optimized the assay and tested the effects of different *B. pertussis* strains and growth conditions on serum and complement sensitivity. We also uncovered complement-independent antibody-mediated inhibition of *B. pertussis* growth. The BGIA can thus effectively be implemented for large-scale serum studies to further investigate anti-*B. pertussis* immune responses at a functional level, as well as for screening of *B. pertussis* strains for their resistance to antibiotics or complement, and for high-throughput screening of novel anti-*B. pertussis* compounds.

## Introduction

Whooping cough or pertussis is a serious and highly contagious respiratory disease mainly caused by the gram-negative bacterium, *Bordetella pertussis* (Hewlett et al., 2014). The disease affects all age groups and is considered as one of the major causes of childhood morbidity and mortality worldwide (Hewlett and Edwards, 2005). In 2014, 24.1 million pertussis cases and 160.700 pertussis-linked deaths were reported in children under the age of 5, among whom 53% were infants younger than 1 year old (Yeung et al., 2017), making this disease the most prevalent vaccine-preventable childhood disease.

The introduction of pertussis vaccination in the 1940s with whole cell pertussis vaccines (wPV) led to a significant decrease in the global pertussis burden. However, due to occasional adverse reactions, acellular pertussis vaccines (aPV) have replaced wPV in the 1990s in most high-income countries. Despite the well-established effectiveness of the both types of current vaccines, and the high global vaccination coverage (Feldstein et al., 2017), there is strong recrudescence of the disease especially in countries using aPV. Substantial vaccine pressure has led to genetic remodeling of strains circulating since the introduction of aPV, particularly of the pertactin gene (Bart et al., 2014). In addition, the emergence of macrolides-resistant strains in China (Wang et al., 2014; Liu et al., 2018; Li et al., 2019) has raised new concerns for transmission and resurgence of pertussis.

Hence, the re-emergence of pertussis is a global public health issue. Therefore, new vaccines that trigger long-lasting and sterilizing immunity to prevent infection and transmission need to be developed (Locht, 2018). In addition, alternative treatments to macrolides in exposed populations should be considered. A better understanding of the pathogenesis of pertussis, protective immunity and drug susceptibility of *B. pertussis* will be useful to define new approaches for the control of this disease.

*B. pertussis* is a tedious organism to culture and requires several days of growth before isolated colonies can be quantified on solid media. This makes high-throughput methods difficult to apply in the context of new anti-*B. pertussis* compound screening or of the analysis of anti-*B. pertussis* immune responses at a functional level. Here, we describe the *Bordetella* growth inhibition assay (BGIA), a luminescence-based method for quantification of surviving bacteria. As it is not based on genetically engineered test organisms, the BGIA can be used on any circulating strain to determine its antibiotic susceptibility and complement resistance, as well as antibody-dependent *B. pertussis* growth inhibition. Results are obtained within hours and the assay is optimized for small volumes making BGIA amendable to high throughput analyses.

## Materials and Equipment

### Bacterial Strains

The streptomycin-resistant Tohama I derivative BPSM (Menozzi et al., 1994), the clinical isolate B1917 (Bart et al., 2010) and three recent pertactin-negative isolates (B1041, B1050 and B1272), generously provided by Dr. Frits R. Mooi (RIVM, Bilthoven, Netherlands), were used to set up the assay.

### Chemicals, Buffers and Media

The pertussis antiserum 06/140 from the National Institute for Biological Standards and Control (NIBSC) was used in this study to develop the BGIA. As exogenous complement sources, IgG- and IgM-depleted human serum (HS), guinea pig serum (GPS) (Sigma) or baby rabbit complement (BRC) (Biorad) were used in the assay. The HS was kindly provided by Andrew Gorringe, Public Health England (PHE). Stock solutions of streptomycin, polymixin b, gentamicin and erythromycin were prepared in water, nalidixic acid in 0.1 M NaOH and chloramphenicol in ethanol were used to determine antibiotic susceptibility. Bacteria and growth inhibitory compounds were diluted in Stainer-Scholte (SS) (Stainer and Scholte, 1971) or Thalen-Ijssel (THIJS) (Thalen et al., 1999).

### Luminometer and Software

Luminescence was read on white half-volume 96 wells plate (Greiner, 675075) by a luminometer (Berthold Centro XS^3^ LB 960) provided with Mikrowin 2000 software. Luminescence was estimated as relative luminescent units (RLU) upon incubation with the BacTiter-Glo reagent (Promega, G8230).

## Materials and Methods

### Bacterial Aliquots Standardization

Bacteria were heavily inoculated on Bordet-Gengou (BG) agar supplemented with 1% glycerol and 10% defibrinated sheep blood and cultured for 2 days at 37°C to obtain a slight lawn. When required, streptomycin was added at 100 μg/ml. Bacteria were then harvested by scraping the plate and resuspending them in phosphate-buffered saline (PBS). The optical density at 600 nm (OD_600_) was adjusted at a final OD_600_ = 0.1 in liquid modified Stainer-Scholte (SS) (Stainer and Scholte, 1971) or Thalen-Ijssel (THIJS) (Thalen et al., 1999) medium. After 16 h of incubation in an orbital-shaking incubator at 200 rpm and 37°C, the log-phased bacteria were harvested by centrifugation at 3,220 × *g* for 10 min at room temperature. The pellet was resuspended with PBS and centrifuged in the same conditions. The washed pellet was resuspended in SS or THIJS supplemented with 15% glycerol at a final concentration of 2.10^6^ colony-forming units CFU/ml. Aliquots of bacteria were stored at −80°C and were added as a ready-to-use liquid-cultured bacteria into the microplate for the BGIA.

### Preparation of Inhibitory Compounds

The pertussis antiserum 06/140, when needed, was decomplemented by heating for 30 min at 56°C to remove endogenous complement activity. The sera were two-fold serially diluted in SS or THIJS media, ranging from 1/2 to 1/4096, and incubated with bacteria suspended in either SS or THIJS media. Alternatively, bacteria were incubated in the presence of HS, GPS or BRC at concentrations ranging from 1 to 15%, diluted in SS or THIJS media. The antibiotics stock solutions were diluted in SS medium at the desired concentrations, and then two-fold serially diluted in SS media. To ensure that the solvent used to dissolve the antibiotics has no effect on bacterial growth, controls containing only the solvents and the bacteria were included in the assay and revealed no significant effect.

### *Bordetella* Growth Inhibition Assay (BGIA)

In each well of a white half-volume 96 wells plate, 10 μl of media or media-containing different amount of antibiotics, complement sources or serum samples was added to 10 μl of aliquoted bacteria. The plate covered with a plastic lid (Greiner, 656190) was centrifuged at 10 × *g* for 1 min and then incubated without shaking at 37°C. After incubation, 20 μl BacTiter-Glo was added to each well, followed by a 10 min incubation at room temperature in an orbital plate shaker. BacTiter-Glo induces bacterial cell lysis for extraction of ATP and production of an ATP-dependent luminescent signal following the reaction between luciferin and luciferase contained within the reagent formulation. After incubation with BacTiterGlo, the luminescence signal was read with a luminometer with the following parameters: 5 min delay by plate and 0.1 sec counting time by well. A first measurement at time 0 (t0), prior to incubation, of the wells containing only bacteria and media was performed to measure the signal corresponding to the initial bacterial inoculum and to monitor aliquot stability.

### Analyses

Raw values expressed in RLU were normalized by calculating the percentages of bacterial survival relative to controls that contained only bacteria and media. Normalized data were plotted against different antibiotic concentrations and complement or serum dilutions to draw a bacterial growth inhibition curve. A four-parameters logistic curve (4PL) on GraphPad Prism5 was applied to normalized data to calculate the half maximal inhibitory concentration (IC_50_) or half maximal inhibitory dilution (ID_50_) with the following equation: *Y* = Bottom + (Top-Bottom)/(1 + 10^[(LogIC50-X)^∗^HillSlope)]. IC_50_ and ID_50_ represent the concentration or the dilution of the inhibitors that induced 50% bacterial growth inhibition. The coefficient of variation (CV) was calculated for each IC_50_ and ID_50_ replicate. The Z’ factor was calculated during the optimization step. Alternatively, the area under the curve (AUC) was measured based on the same growth curves. Statistical analyses were performed with a two-tailed Student’s *t*-test.

## Results

### Correlation Between RLU and CFU Counting

The BGIA developed here is based on bioluminescent detection of ATP produced by living bacteria under different conditions. The luminescence signal is generated through the action of luciferase on luciferin, provided externally by BacTiterGlo, in the presence of ATP provided by metabolically active bacteria. It does not require prior transformation of the bacteria with luciferase-encoding plasmids.

In an attempt to correlate RLU readings with classical CFU counting, different concentrations ranging from 10 to 10^7^ CFU/well of the streptomycin-resistant *B. pertussis* Tohama-I derivative BPSM were seeded in the wells of a white 96 wells plate. Half of the well content was read by luminescence to estimate RLU and the other half was 10-fold serially diluted in PBS and plated onto BG blood agar plates to count CFU upon a 5 days incubation step at 37°C.

**FIGURE 1:**
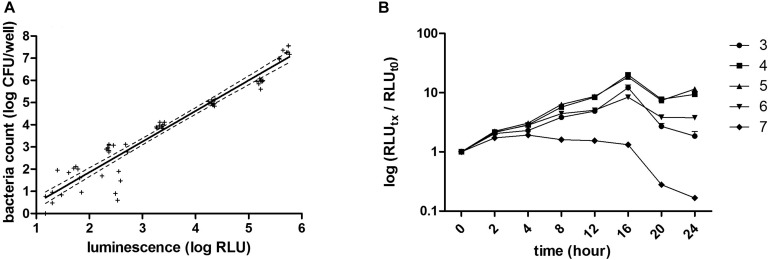
RLU–CFU correlation and optimization of inoculum and culturing time. **(A)** Different bacterial concentrations, ranging from 10^1^ to 10^7^ CFU/well were measured by luminescence (RLU) and by CFU counting. The linear regression [r^2^ = 0.9345, *p* < 0.0001, CFU = 1.37 (±0.05)*RLU – 0.88 (±0.19)] represented by the full line and its deviation indicated by the dotted lines correspond to three independent experiments done for each bacterial concentration in triplicate. **(B)** The bacterial growth kinetic was evaluated by reading luminescence after 0, 2, 4, 8, 12, 16, 20, and 24 h incubation of different concentrations of bacteria, ranging from 10^3^ to 10^7^ CFU/well, represented, respectively, by circles, squares, triangles, reversed triangles and diamonds. Each symbol represents the mean of three independent experiments each done in duplicates. Luminescence read for each time point and for each bacterial concentration was normalized to its corresponding luminescence at time 0.

The intensity of the luminescence signal was proportional to the amount of living bacteria and a good RLU – CFU correlation (r^2^ > 0.93, *p* < 0.0001) was found, especially starting at a concentration of 10^3^ CFU/well and up to 10^7^ CFU/well (Figure 1A).

### Optimization of Inoculum and Culturing Time

Different bacterial concentrations, ranging from 10^3^ to 10^7^ CFU/well, were then incubated for up to 24 h in SS medium and at 0, 2, 4, 8, 12, 16, 20, and 24 h of growth, luminescence was read and normalized to time 0. Luminescence increased over time with the strongest increase seen for cultures seeded at 10^4^ or 10^5^ CFU/well (Figure 1B) up to 16 h of growth. Although an increase in luminescence over time was also seen in wells seeded with 10^3^ or 10^6^ CFU/well up to 16 h of growth, the dynamic range was not as strong as for wells seeded with 10^4^ or 10^5^ CFU/well. From 10^3^ to 10^6^ CFU/well, growth reached a maximal rate at 16 h and then luminescence started to decrease. In wells seeded with 10^7^ CFU/well, the maximal growth was reached after 2 h, and luminescence decreased after 8 h. These results indicate that the inoculum size for *B. pertussis* to obtain the optimal dynamic in this assay is from 10^4^ to 10^5^ CFU/well. They also indicate that *B. pertussis* growth kinetics can be followed over time in a microplate by luminescence reading. The Z’ factors (Zhang et al., 1999), calculated for each bacterial concentration, were −7.3, −4.1, −0.5, 0.6, 0.7, 0.8, and 0.4, corresponding to 10, 10^2^, 10^3^, 10^4^, 10^5^, 10^6^, and 10^7^ CFU/well, respectively. The assay may thus be suitable to examine the effect of compounds or other inhibitors on *B. pertussis* growth over time. Since the growth profiles between 10^4^ and 10^5^ CFU/well inoculum sizes were very similar, we used the 10^4^ CFU/well inoculum size for further assays.

### Antibiotic Susceptibility Tested by BGIA

**FIGURE 2:**
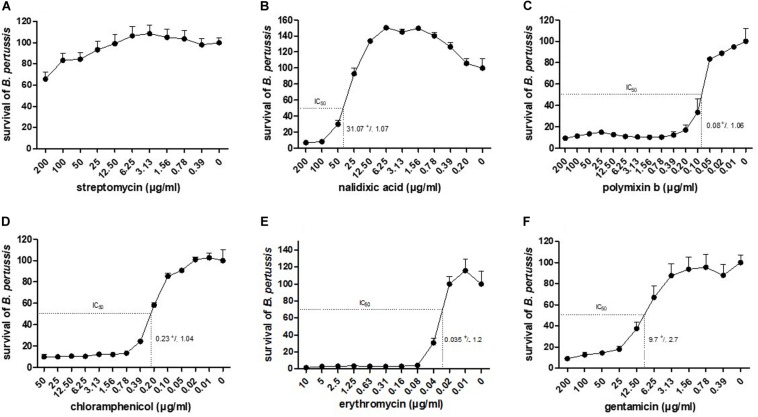
Dose-response curves to antibiotics and corresponding IC_50_. Indicated antibiotics at indicated concentrations were tested for growth inhibition on BPSM. After 16 h of incubation, RLU values are reported in percentages of growth relative to bacterial growth in the absence of antibiotics. The dose-response curves to streptomycin **(A)**, nalidixic acid **(B)**, polymixin b **(C)**, chloramphenicol **(D)**, erythromycin **(E)** and gentamicin **(F)** are represented as means of three independent experiments. The IC_50_ value of each antibiotic is indicated in the corresponding graph.

To assess whether the luminescence measurements can be used to evaluate growth inhibition, various antibiotics were chosen according to their properties and their mechanism of action: (i) the polypeptide polymyxin b and the two aminoglycosides streptomycin and gentamicin, as bacteriolytic antibiotics, (ii) chloramphenicol and a macrolide: erythromycin as bacteriostatic and (iii) nalidixic acid, a quinolone. These antibiotics were incubated at different concentrations with 10^4^ CFU/well of BPSM. To allow for sufficient time for antibiotic action, the incubation was carried out for 16 h. After luminescence reading, the values were normalized to those of the bacteria incubated without antibiotic for 16 h. As BPSM is a streptomycin- and nalidixic acid-resistant derivative of Tohama I, it tolerated high concentrations of these antibiotics (Figures 2A,B). However, at concentrations higher than 25 μg/ml the bacteria were sensitive to nalidixic acid. This allowed us to establish an IC50 at 31.07 ± 1.07 μg/ml for nalidixic acid with a calculated CV of 24%. In contrast to the two former antibiotics, polymyxin b strongly inhibited *B. pertussis* growth even at very low concentrations, the IC_50_ was 0.08 ± 1.06 μg/ml and the CV 3% (Figure 2C). Chloramphenicol (Figure 2D) and erythromycin (Figure 2E) act both by inhibiting protein synthesis but the inhibition profile was different, with a substantially lower IC_50_ for erythromycin than for chloramphenicol, respectively, 0.035 ± 1.2 μg/ml and 0.23 ± 1.04 μg/ml. The calculated CV for chloramphenicol was 5 and 4% for erythromycin. Gentamicin had an IC_50_ (9.7 ± 2.7 μg/ml) higher than chloramphenicol but lower than nalidixic acid (Figure 2F) and a CV of 7%. The calculated IC_50_ determined by the BGIA were compared with the minimal inhibitory concentrations (MIC) determined by standard assays and were found to be in the same range for all antibiotics tested (Table 1). Altogether these data show that the luminescence measurements can be used in a BGIA for testing *B. pertussis* susceptibility to antibiotics and to determine IC_50_ through S-shaped fitted curves (not shown).

**TABLE 1:** BGIA assay’s IC_50_ with standard deviation and MIC from standard assay expressed in μg/ml.

	**Streptomycin (μg/ml ± SD)**	**Nalidixic acid (μg/ml ± SD)**	**Polymyxin b (μg/ml ± SD)**	**Chloramphenicol (μg/ml ± SD)**	**Erythromycin (μg/ml ± SD)**	**Gentamicin (μg/ml ± SD)**
BGIA–IC_50_	>200	31.07 ± 1.07	0.08 ± 1.06	0.23 ± 1.04	0.035 ± 1.2	9.7 ± 2.7
MIC	>800	30–60	0.8–1.6	1–2	0.12–0.25	10–20

### Effect of Serum on *B. pertussis* Growth

**FIGURE 3:**
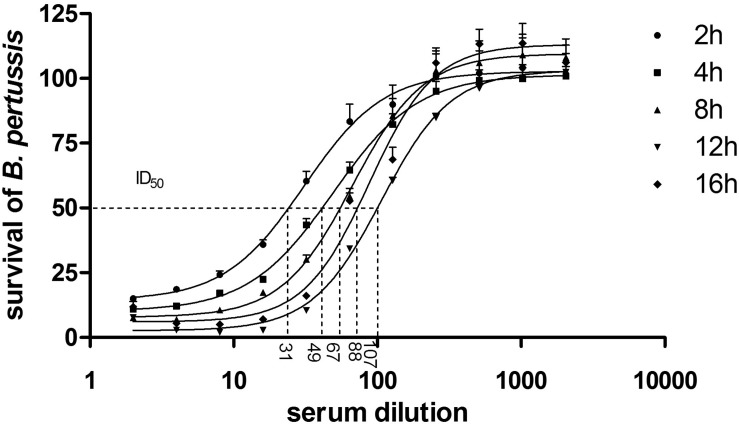
BGIA kinetics using human anti-pertussis serum. 10^4^ CFU/well of BPSM was incubated with two-fold serial dilutions, ranging 1/2–1/2048, of the standard pertussis immune serum NIBSC (06/140). After 2, 4, 8, 12, or 16 h of incubation luminescence was read and normalized for each time point to control bacteria grown without anti-serum. The data are expressed as means of three independent experiments. The non-linear regression curve is represented by the line following a four parameters logistic analysis and the calculated ID_50_ are depicted by the dotted lines with corresponding values for each incubation time that are 30,96 ± 0,07, 49,06 ± 0,03, 67,06 ± 0,04, 106,6 ± 0,01, and 87,70 ± 0,03 after 2, 4, 8, 12, and 16 h, respectively.

To determine whether the luminescence-based BGIA may be useful to analyze the ability of immune serum to restrict *B. pertussis* growth, 10^4^ CFU/well of *B. pertussis* BPSM was incubated with serial two-fold dilutions of the pertussis antiserum NIBSC (06/140), a standard human pertussis immune serum, for 2, 4, 8, 12, or 16 h. After incubation, luminescence was measured and the RLU values were normalized for each time point to control bacteria grown without anti-serum. Plateau values were reached for all time points at the dilution of 1/512 (Figure 3) without significant differences between each incubation time. The S-shaped nature of the curves allowed us to calculate ID_50_s. For short time incubations, the ID_50_ was between the serum dilution 1/16 and 1/32 (for 2 h incubation), and between 1/32 and 1/64 (for 4 h incubation). After 8 h and up to 16 h incubation, the ID_50_ values were similar and ranged between a 1/64 and 1/128 dilution. The calculated CV were 14, 9, 11, 3, and 13% for the ID_50_ after 2, 4, 8, 12, and 16 h incubation, respectively. There was no statistical difference between the growth inhibition profile for 4 or 8 h of serum incubation with *B. pertussis*. Thus, bacterial growth inhibition can be followed at early (2–4 h) and late (8 – 12 – 16 h) time points. We reasoned that bactericidal activity mediated by antibody and complement is a fast-occurring process and therefore carried out all our subsequent experiments with a 4 h incubation time.

### Effect of Complement on *B. pertussis* Growth

**FIGURE 4:**
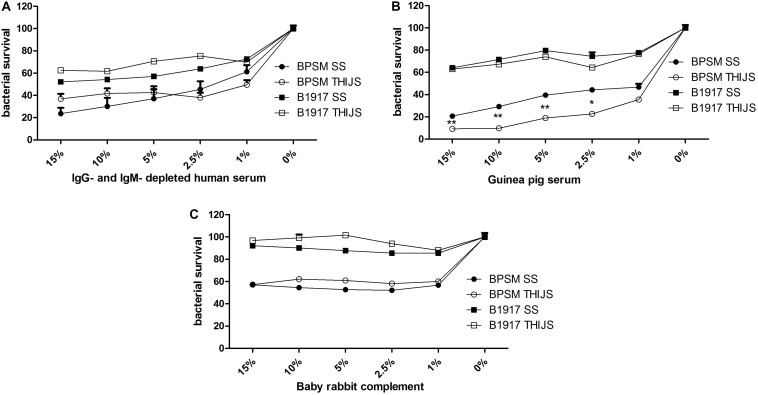
Sensitivity of different *B. pertussis* strains grown in different media to different sources of complement. BPSM (circles) and B1917 (squares) grown in SS (black) or THIJS (white) medium were incubated with different concentrations ranging from 1 to 15% of IgG- and IgM–depleted human serum **(A)**, guinea pig serum **(B)** or baby rabbit complement **(C)**. Luminescence was read after 4 h of incubation and normalized to that of bacteria grown without complement. The results are shown as the means and standard deviations from three independent experiments analyzed by a two-tailed 1-way ANOVA. *p < 0.05, **p < 0.005.

Complement-mediated killing is an important anti-bacterial effector mechanism, and different *B. pertussis* strains may have developed various complement evasion mechanism (for review, see Thiriard et al., 2018). To evaluate the role of complement in *B. pertussis* growth inhibition, we performed the BGIA on IgG- and IgM-depleted human serum (HS), on a commercial guinea pig serum (GPS) and on a baby rabbit complement (BRC). However, sensitivity of *B. pertussis* to complement may vary from strain to strain (Brookes et al., 2018) and according to the growth conditions (Luu et al., 2017). We therefore compared the laboratory strain BPSM to the more recent clinical isolate B1917 each grown in liquid SS or THIJS medium. Different concentrations, ranging from 1 to 15% of HS (Figure 4A), GPS (Figure 4B) and BRC (Figure 4C) were incubated for 4 h with 10^4^ CFU/well BPSM or B1917 grown in SS or in THIJS medium. After normalization of the RLU readings to those of the control bacteria grown in media without complement, for each complement source BPSM appeared more sensitive to complement than B1917. Whereas 30–50% growth inhibition was observed for B1917 in the presence of 1–15% HS, growth inhibition reached 60–80% for BPSM at the same concentrations (Figure 4A). There was no difference between B1917 and BPSM grown in SS or in THIJS medium toward sensitivity to HS. Similarly, growth inhibition reached a maximum of 30–40% with up to 15% of GPS for B1917 and 80–90% growth inhibition with 15% of GPS for BPSM (Figure 4B). Sensitivity to GPS grown in SS or THIJS medium was similar for B1917, whereas BPSM grown in THIJS medium was significantly more sensitive to GPS than BPSM grown in SS medium. Growth inhibition of B1917 mediated by BRC reached a maximum of 10%, whereas inhibition of BPSM growth reached 40% already at a concentration of 1%. No statistical difference between the liquid culture conditions was observed for either strains (Figure 4C). These results indicate that the BGIA can be used to measure differences in complement sensitivity between different *B. pertussis* strains, between growth conditions and between complement sources.

### Complement-Independent and Complement-Dependent Antibody-Mediated Inhibition

**FIGURE 5:**
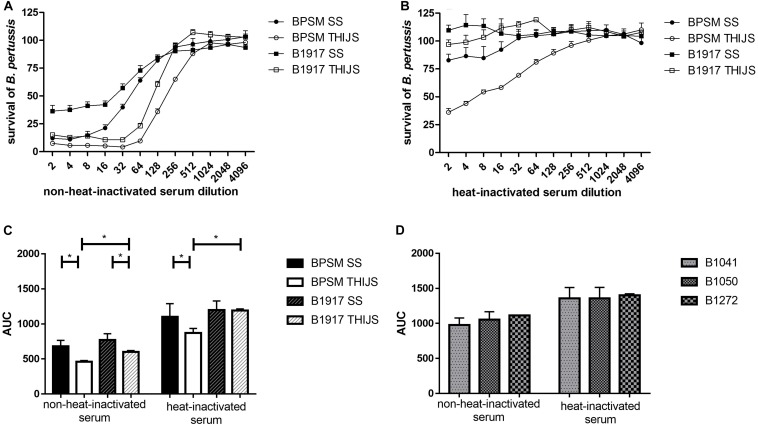
Dose-dependent *B. pertussis* growth inhibition by non-heat-inactivated and heat-inactivated human anti-pertussis serum. BPSM (circles) and B1917 (squares), grown in SS (black) or THIJS (white) medium were incubated for 4 h with two-fold serial dilutions, ranging from 1/2 to 1/4096, with **(A)** NIBSC (06/140) serum or **(B)** heat-inactivated NIBSC (06/140) serum. The luminescence reads were normalized to bacteria grown without antiserum. **(C)** AUC were calculated using the data depicted in Figures 5A,B for BPSM grown in SS medium (black bars) or in THIJS medium (white bars) and B1917 grown in SS medium (gray hatched black filled bars) or THIJS medium (gray hatched white filled bars). **(D)** Calculated AUC of dose-dependent pertactin-deficient B1041 (white bars), B1050 (gray bars) and B1272 (black bars) growth inhibition by non-heat-inactivated and heat-inactivated human NIBSC (06/140) serum, as indicated, after 4 h incubation with two-fold serial dilutions, ranging from 1/2 to 1/4096. Statistical analyses were performed with a two-tailed, Student’s *t*-test on three independent experiments. *p < 0.05, **p < 0.005.

As *B. pertussis* sensitivity to complement can vary between strains, growth conditions and the complement source used, we examined the effect of the standard anti-pertussis serum NIBCS (06/140) on BPSM and B1917 grown either in SS or in THIJS medium. Furthermore, we compared the effect of heat-inactivated NIBCS (06/140) to that of non-heat-inactivated NIBCS (06/140), in order to distinguish between complement-independent and complement-dependent *B. pertussis* growth inhibition, respectively. 10^4^ CFU/well BPSM or B1917 grown in either medium was incubated for 4 h with two-fold serial dilutions of heat-inactivated or non-heat-inactivated NIBCS (06/140) serum, ranging from 1/2 to 1/4096. Both strains grown in either medium were sensitive to NIBCS (06/140) in a dose-dependent manner (Figure 5A). Whereas for BPSM grown in THIJS medium almost 100% growth inhibition was obtained at a 1/32 dilution of NIBCS (06/140), maximum growth inhibition for BPSM grown in SS medium was obtained only at a 1/8 dilution of NIBCS (06/140). The ID_50_ calculated for the growth inhibition curves upon NIBSC (06/140) incubation for BPSM in SS or THIJS media and B1917 in SS or THIJS media were 52 ± 9, 176 ± 20, 47 ± 12, and 125 ± 7, respectively. The calculated CV were 10, 6, 5, and 10%, respectively. For both strains, bacteria grown in THIJS media were more sensitive to complement-dependent antibody-mediated growth inhibition than bacteria grown in SS media. Moreover, while the shape of the inhibition curves was similar between BPSM and B1917 grown in THIJS, they were significantly different between the two strains when grown in SS, showing a higher serum resistance of B1917 than BPSM.

When the NIBSC (06/140) serum was heat inactivated, no growth inhibition was observed for B1917 (Figure 5B) grown in SS or THIJS media. However, slight growth inhibition was observed for BPSM grown in SS medium at the lower serum dilutions, from 1/2 to 1/8. A significantly stronger growth inhibition was seen for BPSM grown in THIJS medium, up to a dilution of 1/128.

The areas under curve (AUC) were calculated for non-heat-inactivated NIBSC (06/140) serum and heat-inactivated serum for each growth condition and bacterial strain, allowing us to quantitatively compare the sensitivity of the two strains grown in the different media (Figure 5C). Growth inhibition mediated by complement-dependent antibody-mediated activity was stronger in THIJS-grown bacteria compared to SS-grown bacteria, and B1917 was significantly more resistant than BPSM when grown in THIJS medium. Significant complement-independent growth inhibition by the antibodies was only observed for BPSM grown in THIJS medium, which was significantly lower than in the presence of complement. Thus, B1917 appeared to be more resistant than BPSM to complement-independent antibody-mediated growth inhibition, in particular when grown in THIJS medium.

Finally, we applied the BGIA to three recent pertactin-deficient clinical isolates. B1041, B1050 and B1272 were grown in SS medium and standardized cultures were then incubated for 4 h with two-fold serial dilutions, ranging from 1/2 to/4096, with the NIBSC (06/140) serum or the heat-inactivated NIBSC (06/140) serum. The luminescence reads were normalized to bacteria grown without antiserum, and AUC were calculated. As shown in Figure 5D, the three pertactin-deficient strains showed sensitivity to heat-inactivated and non-heat-inactivate serum. There was no statistical difference between these three strains. However, a comparison of the data in Figure 5C with those displayed in Figure 5D showed that the pertactin-deficient strains were substantially more resistant to both sera.

## Discussion

Because of the recent outbreaks of *B. pertussis* infections attributed to strain evolution, non-optimal immune responses, fast waning immunity (Mooi et al., 2014) and raising concerns about vaccine- and antibiotic-resistance, development of robust assays to assess induced antibody functionality and antibiotic susceptibility may be helpful to develop improved vaccines and treatments. However, existing assays rely on CFU counting as readout of bacterial survival, which is tedious, time-consuming and labor-intensive, thus making this readout difficult to apply for large-scale studies or high-throughput screening.

In this study, we propose a scalable high-throughput assay based on luminescence readout, as an alternative to the classical CFU counting to assess bacterial growth inhibition. The BGIA is sensitive, reproducible, fast and easy-to-operate and was optimized for small volume samples. We demonstrate here that *B. pertussis* is able to grow in microplates and that growth can be followed over time by luminescence reading. We also demonstrate that the BGIA can be used to measure *B. pertussis* growth in the presence of antibiotics, complement or immune serum and document the effect of the bacterial growth conditions and strains in complement-dependent and –independent antibody-mediated growth inhibition. Strengths of this assay over previously described luminescence-based assays resides in the fact that the BGIA described here is fast operating, without the need of freshly liquid-cultured bacteria, and can be easily used on any *B. pertussis* isolate, without prior need for transformation with luciferase-encoding plasmids, as the enzyme and substrate, except for ATP, are exogenously provided by BacTiter-Glo.

While a very strong correlation was found between CFU counting and RLU measurements (r^2^ = 0.9345, *p* < 0.0001), both assays do not necessarily measure the same effects of the antibacterial compounds. Even though growth inhibition can be followed over time by OD reading to provide information on lytic and static inhibitor effects, a second step of CFU plating and counting on solid media, after the incubation with anti-bacterial compounds, is required to measure bactericidal activities of these compounds. In contrast, the BGIA described here measures both bactericidal and bacteriostatic activities of anti-bacterial compounds in a one-step protocol. As this BGIA is an ATP-based assay with a luminescent readout to measure bacterial growth by the ATP content, an increase or decrease in luminescence is the result of an increase or decrease in ATP levels produced by metabolically active bacteria. This therefore indicates bacterial growth or growth inhibition. Unlike CFU-based methods or OD measurements, this luminescence-based assay can be conveniently used to follow bacterial growth over time, without the need for a second step of bacterial growth on solid media.

The protocol of the BGIA described here is simple and requires no washing step or medium removal. It is fast, as direct luminescence acquisition requires less than 2 min per plate. It is highly reproducible and sensitive, as the CV of the calculated ID_50_ or IC_50_ were usually low. The BGIA is performed in a white half-area 96-wells microplate to reduce light crosstalk between neighboring wells. Ideally, luminescence should be read on the top of the plate with a microplate reader (Berthold, Centro XS3 LB 960) providing better signal-to-noise ratios and sensitivity compared to a bottom reading (BMG Labtech, POLARstar Omega) (data not shown). The Z’factor corresponding to 10^4^ CFU/well was >0.5, indicating that the BGIA is robust for high-throughput analysis. Moreover, the BGIA can be performed in small volumes, i.e., 10 μl per well of tested antibacterial compounds. This is particularly interesting for testing of precious serum samples, as the amount of serum is often a limiting factor in human and animal studies. Although this study describes results obtained with 96-well plates, for some experiments we have also used 384-well plates with similar results (data not shown). This luminescence-based assay makes use of the commercially available BacTiter-Glo, which contains standardized reagents enabling an enzymatic reaction catalyzed by a recombinant firefly luciferase between a beetle luciferin and intracellular ATP extracted by a non-ionic surfactant. This reaction generates oxyluciferin and photon emission that directly depends on ATP input and therefore is proportional to the number of viable and metabolically active bacteria. As the bacterial metabolism strongly depends on culture conditions and media used, we have standardized them and found that different strains (e.g., BPSM and B1917 used in this study) may behave differently when grown in two different media, such as the SS and the THIJS media. As the BGIA can conveniently be used to compare serum samples for their bacterial growth inhibition properties, it is therefore essential that culture conditions are carefully controlled. Importantly, neither serum nor compounds present in SS or THIJS media appear to interfere with luminescence reading (data not shown).

After standardization we found that inoculum sizes of *B. pertussis* ranging from 10^3^ to 10^6^ CFU/well were able to multiply 10-fold over 12–16 h incubation, whereas with an inoculation of 10^7^ CFU/well growth stopped already after only 2 h of incubation. Inoculum sizes between 10^4^ and 10^5^ CFU/well appeared to be ideal to measure growth inhibition by antibiotics, complement and serum samples, suggesting an optimal bacterial concentration input to reach the best growth dynamic. Although there is so far no evidence of quorum sensing in *B. pertussis*, sensing of general changes in local conditions, such as nutrient depletion and secretion of inhibitory molecules by *B. pertussis*, can lead to growth rate adjustment (Nakamura et al., 2006) and differences in virulence factor secretion (Hozbor et al., 1993; Westdijk et al., 1997; Bogdan et al., 2001). Therefore, the growth phase and inoculum size are important parameters to take into account for the evaluation of growth inhibiting compounds.

We also used the BGIA to measure sensitivity/resistance of *B. pertussis* to antibiotics. While antibiotic-resistant *B. pertussis* strains were rare in the past, resistance to the drugs routinely used to treat pertussis has emerged in recent decades. The first erythromycin-resistant strains were isolated in the United States (Lewis et al., 1995; Korgenski and Daly, 1997) in the 1990s and outbreaks of macrolide-resistant strains were reported with increased frequency in China (Wang et al., 2014; Yang et al., 2015; Liu et al., 2018; Li et al., 2019). A few erythromycin-resistant strains were also isolated in Iran (Shahcheraghi et al., 2014) and France (Guillot et al., 2012). In these studies, antibiotic resistance had been assessed by agar-based enumeration methods (Hill et al., 2000), which could easily be replaced by the luminescence-based BGIA, allowing for the testing of sensitivity/resistance to several antibiotics simultaneously. BPSM is a streptomycin- and nalidixic acid-resistant strain. However, high concentrations of these antibiotics, above 25 μg/ml, inhibited *B. pertussis* growth, especially for nalidixic acid. At low concentrations, both antibiotics are known to be bacteriostatic, while they are bactericidal at high concentrations, even though their mechanism of action is different, as streptomycin binds to the 16S RNA and nalidixic acid inhibits the bacterial DNA gyrases and Topoisomerases IV (Correia et al., 2017). The resistance mechanism toward nalidixic acid has not been extensively studied in *B. pertussis* but according to the growth inhibition curve profile, a saturation point of the quinolone is reached earlier, above 25 μg/ml leading to high sensitivity (IC_50_ = 31.07 ± 1.07 μg/ml), compared to streptomycin. As a cationic peptide that binds cell membrane to drill pores at the surface, polymyxin b is highly efficient against *B. pertussis* growth and IC_50_ = 0.08 ± 1.06 μg/ml could be calculated, and chloramphenicol that inhibits protein synthesis by binding to the 23S RNA had an IC_50_ of 0.23 ± 1.04 μg/ml. Erythromycin, an antibiotic used against *B. pertussis*, is a macrolide, which is now often replaced by azithromycin, clarithromycin or trimethoprim-sulfamethoxazole due to emergence of erythromycin-resistance. The IC_50_ of BPSM toward erythromycin was found to be around 0.035 ± 1.2 μg/ml as measured by the BGIA. The values obtained by the BGIA were in the same range as those determined by standard MIC assays and were also consistent with concentrations described in *B. pertussis* literature (Field and Parker, 1980; Bannatyne and Cheung, 1982). Thus, this BGIA can be used on clinical isolates to assess rapidly their antibiotic resistance in order to adapt antibiotic treatments and thus impede the spread of resistant strains.

*B. pertussis* has developed several complement evasion strategies to subvert host innate immunity (Thiriard et al., 2018), and complement sensitivity may vary among different clinical isolates (Stefanelli et al., 2006; Brookes et al., 2018) and depends on the secretion of various virulence factors that can be modulated during growth (Barnes and Weiss, 2002) and culture conditions (Luu et al., 2017). In this study, we show that the BGIA can be used to measure complement sensitivity and confirm that sensitivity differs between strains, bacterial growth medium and complement source. As such, B1917 is more resistant to complement than BPSM, and for BPSM, but not for B1917, sensitivity to guinea pig complement was higher when the bacteria were grown in THIJS medium compared to growth in SS medium. No difference between bacteria grown in SS or THIJS media was seen when the sensitivity to human or mouse complement was tested. Thus, according to the source of complement used to assess complement-resistance, susceptibility may vary. Non-standardized assays to evaluate *B. pertussis* immune serum-sensitivity may explain some conflicting reports due to complement diversity and final concentrations used. Furthermore, serum resistance is *bvg*-regulated (Fernandez and Weiss, 1998; Marr et al., 2007) and can be attributed to the *Bordetella* resistance killing antigen (BrkA) (Fernandez and Weiss, 1994; Elder and Harvill, 2004) and to the virulence-associated gene 8 (Vag8) (Passerini De Rossi et al., 1999; Marr et al., 2011). BGIA could also be used to test different *B. pertussis* strains deficient in *bvg*-regulated-genes to decipher more precisely which virulence genes are associated to complement-resistance.

The BGIA also shows differences among strains and culture conditions with respect to sensitivity to immune serum, both heat-inactivated and non-heat-inactivated. BPSM was generally more sensitive than B1917 and bacteria grown in THIJS medium were generally more sensitive to human immune serum than bacteria grown in SS. Interestingly, heat-inactivated serum inhibited only BPSM growth when the strain was cultured in THIJS medium. The functional properties of heat-inactivated serum are mediated by antibodies independently of complement. Interestingly, when recent pertactin-deficient clinical isolates were tested, they were found to be more resistant to heat-inactivated and non-heat-inactivated serum than the two other strains. These findings suggest that pertactin may be a major target for human immune serum. Since the NIBSC (06/140) serum is a pool of human convalescent and aPV-induced sera, it is possible that anti-pertactin antibodies (together with anti-pertussis toxin and anti-filamentous hemagglutinin antibodies) predominate in the NIBSC (06/140) serum.

The correlates of vaccine- or infection-induced immunity to pertussis are still ill-defined, as, with the exception of pertussis toxin-neutralization assays, most studies focused on serum immunoglobulin levels rather than on functional studies of immune sera. In order to decipher immune correlates of protection against infection or disease the BGIA may thus be a useful tool to establish immunological signatures of protection via antibody functionality studies.

## Data Availability Statement

All datasets generated for this study are included in the article/supplementary material.

## Author Contributions

AT, DR, and CL conceived and designed the experiments, contributed materials and analysis tools, performed the data analysis, and wrote the manuscript. AT performed the experiments.

## Conflict of Interest

The authors declare that the research was conducted in the absence of any commercial or financial relationships that could be construed as a potential conflict of interest.
